# Liver fibrosis is associated with left ventricular remodeling: insight into the liver-heart axis

**DOI:** 10.1007/s00330-024-10798-1

**Published:** 2024-05-25

**Authors:** Carl Edin, Mattias Ekstedt, Markus Karlsson, Bertil Wegmann, Marcel Warntjes, Eva Swahn, Carl Johan Östgren, Tino Ebbers, Peter Lundberg, Carl-Johan Carlhäll

**Affiliations:** 1 Division of Diagnostics and Specialist Medicine, Department of Health, Medicine and Caring Sciences, Linköping University, Linköping, Sweden; 2 Center for Medical Image Science and Visualization (CMIV), Linköping University, Linköping, Sweden; 3 Department of Clinical Physiology in Linköping, and Department of Health, Medicine and Caring Sciences, Linköping University, Linköping, Sweden; 4 Department of Radiation Physics, and Department of Health, Medicine and Caring Sciences, Linköping University, Linköping, Sweden; 5 Department of Computer and Information Science, Linköping University, Linköping, Sweden; 6 Department of Cardiology in Linköping, and Department of Health, Medicine and Caring Sciences, Linköping University, Linköping, Sweden; 7 Division of Prevention, Rehabilitation and Community Medicine, Department of Health, Medicine and Caring Sciences, Linköping University, Linköping, Sweden

**Keywords:** Interleukin-1, Non-alcoholic fatty liver disease, Type 2 diabetes, Elastography, Magnetic Resonance

## Abstract

**Objective:**

In nonalcoholic fatty liver disease (NAFLD), liver fibrosis is the strongest predictor of adverse outcomes. We sought to investigate the relationship between liver fibrosis and cardiac remodeling in participants from the general population using magnetic resonance imaging (MRI), as well as explore potential mechanistic pathways by analyzing circulating cardiovascular biomarkers.

**Methods:**

In this cross-sectional study, we prospectively included participants with type 2 diabetes and individually matched controls from the SCAPIS (Swedish CArdioPulmonary bioImage Study) cohort in Linköping, Sweden. Between November 2017 and July 2018, participants underwent MRI at 1.5 Tesla for quantification of liver proton density fat fraction (spectroscopy), liver fibrosis (stiffness from elastography), left ventricular (LV) structure and function, as well as myocardial native T1 mapping. We analyzed 278 circulating cardiovascular biomarkers using a Bayesian statistical approach.

**Results:**

In total, 92 participants were enrolled (mean age 59.5 ± 4.6 years, 32 women). The mean liver stiffness was 2.1 ± 0.4 kPa. 53 participants displayed hepatic steatosis. LV concentricity increased across quartiles of liver stiffness. Neither liver fat nor liver stiffness displayed any relationships to myocardial tissue characteristics (native T1). In a regression analysis, liver stiffness was related to increased LV concentricity. This association was independent of diabetes and liver fat (Beta = 0.26, *p* = 0.0053), but was attenuated (Beta = 0.17, *p* = 0.077) when also adjusting for circulating levels of interleukin-1 receptor type 2.

**Conclusion:**

MRI reveals that liver fibrosis is associated to structural LV remodeling, in terms of increased concentricity, in participants from the general population. This relationship could involve the interleukin-1 signaling.

**Clinical relevance statement:**

Liver fibrosis may be considered a cardiovascular risk factor in patients without cirrhosis. Further research on the mechanisms that link liver fibrosis to left ventricular concentricity may reveal potential therapeutic targets in patients with non-alcoholic fatty liver disease (NAFLD).

**Key Points:**

- *Previously, studies on liver fibrosis and cardiac remodeling have focused on advanced stages of liver fibrosis.*
- *Liver fibrosis is associated with left ventricular (LV) concentricity and may relate to interleukin-1 receptor type 2.*
- *Interleukin-1 signaling is a potential mechanistic interlink between early liver fibrosis and LV remodeling.*

## Introduction

Non-alcoholic fatty liver disease (NAFLD, also termed *metabolic dysfunction-associated steatotic liver disease—*MASLD), estimated to affect more than half of the global type 2 diabetes population [1], has emerged as a risk factor for cardiac remodeling and development of heart failure [2–4]. Although the mechanisms are not yet fully understood, the underlying degree of hepatic fibrosis in NAFLD has been linked to both higher mortality in acute heart failure [5] and left ventricular (LV) diastolic dysfunction [6, 7].

To date, however, research on liver fibrosis and cardiac remodeling has focused on patients from tertiary centers with advanced liver scarring (i.e., cirrhosis) [8–10]. This is despite the fact that NAFLD is typically a long-term progressive disease, where cirrhosis is preceded by several years of steatosis, inflammation, and subclinical fibrosis in the liver [11]. Few studies have reported on the relationship between liver fibrosis and cardiac remodeling in patients free of cirrhosis. Moreover, none of these studies has used magnetic resonance imaging (MRI), a method that provides accurate and non-invasive measurements of both hepatic fat content and fibrosis [12], as well as enabling detailed assessment of cardiac function, structure, and tissue characteristics [13, 14].

In order to investigate the relationships between liver fibrosis and cardiac remodeling in the early stages of fatty liver disease, we applied advanced cardiac and hepatic magnetic resonance techniques in participants sampled from the general population. The aim was to assess the relationships between liver fibrosis and cardiac structure, function, and tissue characteristics. We also sought to explore potential underlying mechanistic pathways by analyzing circulating biomarkers of cardiovascular inflammation and fibrosis. The hypothesis was that liver fibrosis had a significant association to LV structure, function, and tissue characteristics.

## Methods

### Study design and population

The study was approved by the Regional Ethics Committee Linköping and conducted in accordance with the Declaration of Helsinki. All participants provided written informed consent to participate.

The current study was a prospective cross-sectional sub-study within SCAPIS (Swedish CArdioPulmonary bioImage Study). SCAPIS is a nation-wide cohort study including over 30,000 men and women in the ages 50–65, all randomly sampled from the general Swedish population [15]. As described previously [16], we consecutively recruited participants with type 2 diabetes (T2D) as well as controls free of diabetes from the SCAPIS cohort in Linköping (*n* = 5 000). Participants were identified as having diabetes through SCAPIS health forms, having fasting P-glucose ≥ 7.0 mmol/L on two occasions or HbA1C ≥ 48 mmol/mol by sampling at SCAPIS. Controls were individually matched to the diabetes participants with respect to age, sex, and smoking habits. Participants with type 1 diabetes, general contraindications to MRI, or significantly irregular ventricular rhythm were excluded.

### SCAPIS data

All subjects in this study underwent the core SCAPIS data acquisition. Accordingly, we had access to the following data for our study population: demographics (age, sex), alcohol consumption and smoking, self-reported cardiac disease (coronary heart disease, atrial fibrillation, heart failure or valvular heart disease), drug use, anthropometrics (height, weight, and body mass index [BMI]), clinical biochemistry and echocardiography (diastolic functional parameters).

See Supplementary material for additional information on the acquisition and analysis of echocardiographic data.

### Magnetic resonance

Between November 2017 and July 2018, and within four weeks after the core SCAPIS data collection, all participants underwent a magnetic resonance scan at Linköping University Hospital. Data were acquired in a single scan at 1.5 T using Philips Achieva dStream (Philips Healthcare). The scan covered several sequences, e.g., for quantification of liver fat, liver stiffness, LV structure, and function, as well as LV tissue characteristics.

#### Liver

##### Elastography

**Fig. 1:**
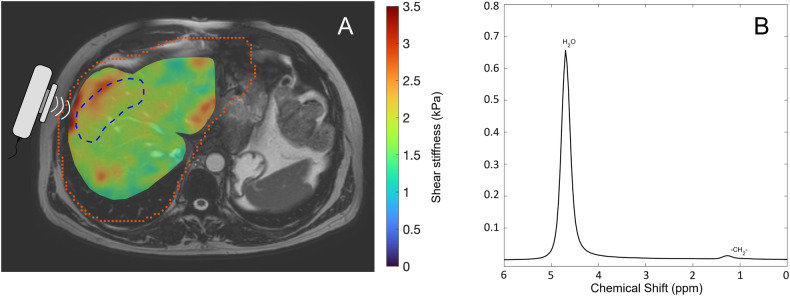
**A** Liver elastogram from magnetic resonance elastography (3D MRE) in a 53-year-old male in the diabetes group, with a liver shear stiffness of 2.2 kPa. The position of the external transducer on the right anterior chest wall and the axial waves are schematically illustrated. The region of interest (ROI-2) for measurement of shear stiffness, located in the right liver lobe, is shown in blue (dashed). The larger region of interest (ROI-1) for defining the input region to the KIR-software is shown in orange (dotted), and the coloured overlay shows the result of the calculation within the region representing liver, on a background anatomical image. The image is presented using radiological convention. **B** Liver proton spectra from magnetic resonance spectroscopy, used for proton density fat fraction (PDFF) calculation, with water (H_2_O) resonance at 4.76 ppm and major fatty-acyl chain (-CH_2_-) resonance at 1.21 ppm

As a metric of fibrosis in the liver, liver stiffness was measured using 3D magnetic resonance elastography (MRE). The procedure was performed as previously described [17]. In short, an active electrodynamic transducer at 56 Hz was used, positioned to the right of the xiphoid process on the anterior chest wall, and held firmly in place using a broad elastic band. From four breath holds, a 3D image was acquired with nine slices, each with a slice thickness of 4 mm. Mean shear stiffness was determined using the KIR-software (for 3D processing), originally described in [18], and the results were reported in kPa. Regions of interest for the ROI measurements were defined away from large vessels and organ edges and drawn in the right liver lobe. See Fig. 1 for an example liver MR (magnetic resonance) elastogram.

##### Spectroscopy

Liver proton density fat fraction (PDFF) was measured using magnetic resonance spectroscopy (MRS), as described in previous publications [16, 19, 20]. In short, data was acquired with a ^1^H-MRS PRESS sequence and subsequently post-processed to quantify the integrals of water and fat resonance, using the jMRUI and AMARES algorithms, respectively [21, 22]. Regions of interest of 30 × 30 × 30 mm^3^ were placed in the right liver lobe, in an area free from major blood vessels. All resonances from different lipid moieties were included in the spectral analysis. See Fig. 1 for an example liver spectrum from the MRS sequence.

#### Cardiac

##### Cine imaging

**Fig. 2:**
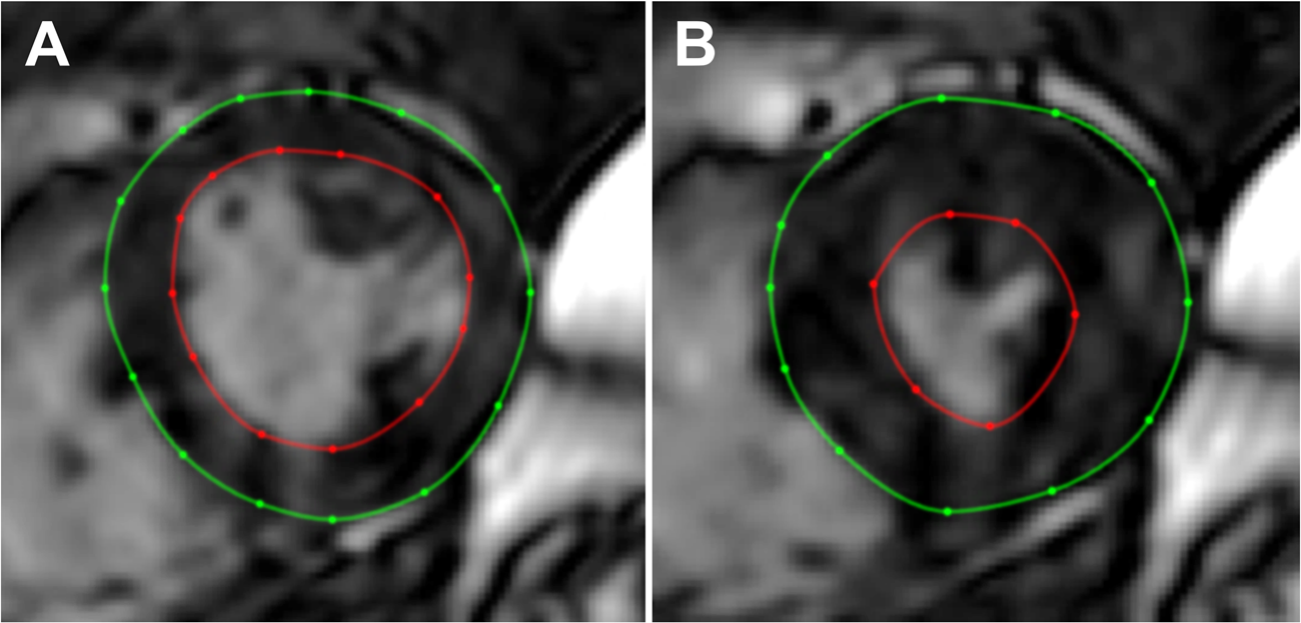
Mid-ventricular short-axis image of the left ventricle at end-diastole (**A**) and end-systole (**B**) in a participant with an LV ejection fraction of 66% and LV concentricity ratio of 1.06 g/mL

A 3D cine balanced steady state-free precession sequence was used to assess LV structure (mass, end-diastolic volume, concentricity) and function (ejection fraction, stroke volume). Images were acquired during breath hold and subsequently post-processed by a reader (CE) with five years of cardiac imaging experience using Segment CMR version 3.2 R8452 (Medviso AB) [23]. LV mass was acquired in end-diastole, and the papillary muscles were regarded as part of the blood pool. LV concentricity was calculated as the ratio between LV mass and LV end-diastolic volume. Cardiac MR has previously shown excellent reproducibility for LV mass and volumes [24]. Where applicable, the Mosteller algorithm [25] was used to index the LV parameters and left atrial volume to body surface area (BSA): BSA (m^2^) = (height (cm) × weight (kg)/3600)^0.5^. See Fig. 2 for example images from the cardiac cine sequence.

##### T1 and T2 mapping

**Fig. 3:**
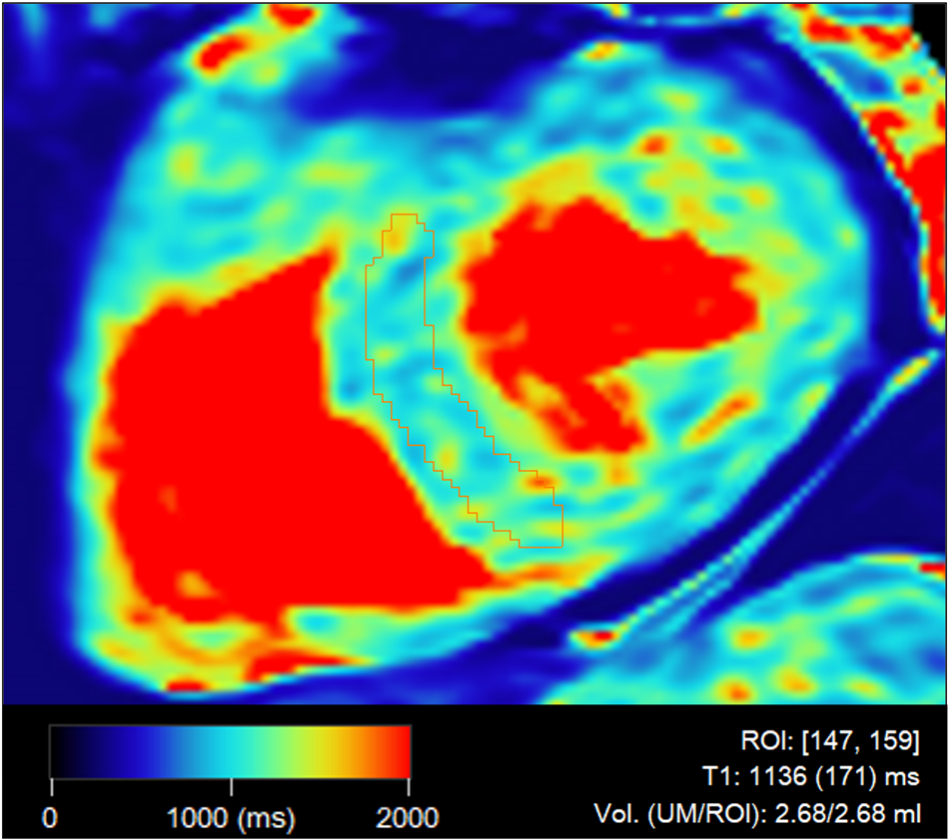
Quantitative native T1 map, in a mid-ventricular slice from the 3D-QALAS sequence. The region of interest is shown in red, in the septal myocardium

Quantitative mapping of native T1 and T2 of the heart was performed using 3D-QALAS (3D-quantification using an interleaved Look-Locker sequence with T2 preparation pulse), enabling three-dimensional mapping in the whole heart during a single breath hold [26, 27]. Quantitative maps were generated and post-processed using software SyMRI version 0.45.38 (SyntheticMRI AB). All datasets were assessed by C.E. Ten datasets were randomly selected for inter- and intrareader variability analysis and assessed by E.K. (two years of cardiac imaging experience), as well as a second time by C.E. 8 weeks after the first assessment. As proposed by Puntmann et al [28], regions of interest were drawn manually in a short axis view, in the septal wall of all 3 to 5 mid-ventricular slices where the papillary muscles of the LV were visible. In order to avoid border artefacts towards the blood pool, only the innermost ~50% of the thickness of the septal myocardium was included. Average T1 and T2 values were calculated from all slices. See Fig. 3 for an example image from the 3D-QALAS sequence.

### Proteomics

Blood samples were collected during the core SCAPIS data collection and accessible through the Linköping bio-bank facility. Three commercially available biomarker panels were analyzed externally using proximity extension assay technology: Cardiovascular II, Cardiovascular III, and Cardiometabolic (Olink Proteomics AB), totaling 276 unique biomarkers for cardiovascular inflammation, metabolism, and remodeling. In addition, PRO-C6, a marker of fibroblast activity, was also analyzed externally (Nordic Bioscience). A linear scale was used for all biomarkers.

### Statistical analyses

#### Group comparisons and linear regression analyses

Statistical comparison of groups and quartiles, as well as multiple linear regression analyses, were performed in IBM SPSS version 29. Independent samples T-tests were used to compare continuous data between the diabetes and the control group. Chi-square tests were used to compare categorical data between the two groups. To compare cardiac parameters between quartiles of liver stiffness and liver PDFF, we used the Kruskal-Wallis test for all parameters due to *n* < 30 in each quartile. Intra- and interreader variability was assessed by single measures intraclass correlation coefficient (ICC), using the absolute-agreement two-way mixed-effect model. Pearson correlation coefficients were used to assess the relationships of cardiac remodeling parameters to clinical liver biochemistry. Multiple linear regression was used to assess linear relationships between cardiac remodeling and hepatic MRI parameters as well as to cardiovascular proteomics. Due to left-skewness, the liver fat percentage was logarithmically transformed for the linear regression analyses. For all regression models, the normal distribution of residuals was affirmed using Shapiro-Wilks test. For each model, we calculated the *p* value and standardized beta coefficient for each independent variable, as well as the overall *R*^2^ value. *p* values < 0.05 were considered statistically significant.

#### Stochastic search variable selection

To explore the relationships between the circulating cardiovascular proteomics and measures of LV remodeling, we used the Stochastic Search Variable Selection (SSVS) [29, 30]. SSVS is a Bayesian statistical method that, for a given dependent variable, efficiently samples a smaller subset of predictors from a larger set of predictors. This is done by stochastically iterating over thousand several subsets of predictors to simultaneously model the strength of predictors and uncertainty in the regression parameters. The proportion of times each predictor is included in the model, the ‘posterior inclusion probability’, can be used to assess the relative strength for each predictor. The closer the posterior inclusion probability is to 1, the stronger a predictor is to explain the dependent variable.

SSVS was performed by BW using the R package *SSVS*. Four models were estimated, each with a different dependent variable: LV concentricity, LV end-diastolic volume, LV mass and LV E/é. All biomarkers were used in all four models. We selected a standard choice of non-informative prior distributions, with a prior inclusion probability of 0.5 for each predictor. As an analogous interpretation of Bayes factors [31], a posterior inclusion probability above ~0.76 was considered *substantial* and above ~0.91 was considered *strong*. Less-than-*substantial* predictors were not considered. Beta coefficients were standardized, to enable comparison between predictors.

## Results

### Clinical and biochemical data

92 participants were enrolled (mean age 59.5 ± 4.6 years, 32 women); 46 with type 2 diabetes and 46 controls without diabetes. 11 participants (12.0%) reported previous cardiac disease. The average alcohol consumption was 45 g/week. One participant reported excessive alcohol consumption (> 210 g/week). Women had higher high-density lipoprotein (HDL) cholesterol than men (1.8 vs. 1.3 mmol/L, *p* < 0.001), otherwise there were no sex differences in clinical and biochemical data.

The diabetes group had higher BMI (29.6 vs. 26.2 kg/m^2^, *p* < 0.001), higher HbA1c (54 vs. 34 mmol/mol, *p* < 0.001), and higher alanine aminotransferase (ALT) (0.70 vs 0.50 µkat/L, *p* = 0.017) compared to the control group. 10 participants in the diabetes group (21.7%) had insulin treatment (Table 1).

**Table 1:** Clinical and biochemical data

	Total (*n* = 92)	T2D (*n* = 46)	Controls (*n* = 46)	*p* value (T2D vs. controls)
Age (years)	59.5 ± 4.6	59.6 ± 4.6	59.3 ± 4.7	0.79
Sex (women)	32 (34.8%)	16 (34.8%)	16 (34.8%)	–
Current smoking	8 (8.7%)	4 (8.7%)	4 (8.7%)	–
Alcohol consumption (g/week)	45 ± 49	38 ± 32	52 ± 61	0.17
Self-reported cardiac disease^a^	11 (12.0%)	8 (17.4%)	3 (6.5%)	0.11
Time since diabetes diagnosis (years)	–	6.4 ± 5.5	–	N/A
Insulin use	–	10 (21.7%)	–	N/A
Anti-hypertensive medication	28 (30.5%)	23 (50%)	5 (10.9%)	< 0.001
Lipid-lowering medication	22 (23.9%)	19 (41.3%)	3 (6.5%)	< 0.001
Weight (kg)	84.8 ± 13.6	88.9 ± 13.7	80.7 ± 12.3	0.0034
Height (m)	1.74 ± 0.09	1.73 ± 0.09	1.75 ± 0.09	0.21
BMI (kg/m^2^)	27.9 ± 3.7	29.6 ± 3.3	26.2 ± 3.2	< 0.001
Heart rate (BPM)	69 ± 12	73 ± 13	66 ± 10	0.0055
Systolic blood pressure (mm Hg)	140 ± 17	144 ± 15	137 ± 17	0.071
Diastolic blood pressure (mm Hg)	84 ± 12	86 ± 13	82 ± 10	0.10
HbA1c (mmol/mol)	44 ± 15	54 ± 16	34 ± 3	< 0.001
Total cholesterol (mmol/L)	5.0 ± 1.4	4.3 ± 1.1	5.7 ± 1.2	< 0.001
LDL cholesterol (mmol/L)	2.9 ± 1.2	2.2 ± 1.0	3.6 ± 1.0	< 0.001
HDL cholesterol (mmol/L)	1.5 ± 0.5	1.4 ± 0.5	1.6 ± 0.5	0.020
Triglycerides (mmol/L)	1.4 ± 0.9	1.7 ± 1.1	1.2 ± 0.6	0.0086
hsCRP (mg/L)	2.1 ± 2.5	2.0 ± 2.2	2.1 ± 2.8	0.81
ALT (µkat/L)	0.60 ± 0.42	0.70 ± 0.46	0.50 ± 0.35	0.017
AST (µkat/L)	0.50 ± 0.23	0.51 ± 0.19	0.49 ± 0.26	0.61
GGT (µkat/L)	0.76 ± 0.67	0.91 ± 0.76	0.62 ± 0.53	0.039
Creatinine (µmol/L)	80 ± 16	76 ± 16	84 ± 15	0.015
NT-proBNP (ng/L)	75 ± 99	72 ± 103	78 ± 96	0.79

Groups comparison of clinical and biochemical data. Continuous data are shown as mean ± SD and categorical data as n (%). *LDL* low-density lipoprotein, *hs-CRP* high-sensitivity C-reactive protein, *GGT* gamma-glutamyl transferase, *NT-proBNP* N-terminal pro–B-type natriuretic peptide, *AST* asparate aminotransferase

^a^ coronary heart disease, atrial fibrillation, heart failure, or valvular heart disease

### Magnetic resonance and echocardiography data

The mean liver PDFF for all 92 subjects was 8.7%, where the diabetes group displayed higher liver PDFF than the control group (11.7 vs. 5.7%, *p* < 0.001). A total of 53 participants (57.6%) displayed hepatic steatosis defined as liver PDFF ≥ 5%, 33 (71.7%) in the diabetes group and 20 (43.5%) in the control group (*p* = 0.011 for group difference). The mean liver stiffness value was 2.1 kPa in the diabetes group and 2.0 kPa in the control group (*p* = 0.062 for group difference). The highest observed liver stiffness value was 2.9 kPa.

Compared to the control group, the diabetes group had lower LV end-diastolic volume (69.6 vs. 76.0 mL/m^2^, *p* = 0.047), higher LV concentricity (0.91 vs. 0.79 g/mL, *p* < 0.001), and higher LV E/é ratio (13.0 vs. 9.9, *p* < 0.001). There were no statistically significant differences in LV ejection fraction, LV stroke volume, LV mass, septal T1, septal T2, left atrial volume, or LV E/A ratio (Table 2).

**Table 2:** Magnetic resonance and echocardiography data

	Total (*n* = 92)	T2D (*n* = 46)	Controls (*n* = 46)	*p* value (T2D vs controls)
**Liver parameters**				
Liver PDFF (%)	8.7 ± 8.5	11.7 ± 9.0	5.7 ± 6.8	< 0.001
Liver steatosis (PDFF > 5%)	53 (57.6%)	33 (71.7%)	20 (43.5%)	0.011
Liver stiffness (kPa)	2.1 ± 0.4	2.1 ± 0.4	2.0 ± 0.4	0.079
**Cardiac parameters**				
LV ejection fraction (%)	59.3 ± 8.8	59.9 ± 9.2	58.6 ± 6.9	0.45
LV stroke volume (mL/m^2^)	42.5 ± 8.8	40.9 ± 9.1	44.2 ± 8.3	0.076
LV mass (g/m^2^)	60.6 ± 11.3	62.2 ± 12.5	59.0 ± 9.7	0.17
LV end-diastolic volume (mL/m^2^)	72.8 ± 15.6	69.6 ± 15.2	76.0 ± 15.6	0.047
LV concentricity (g/mL)	0.85 ± 0.14	0.91 ± 0.12	0.79 ± 0.12	< 0.001
Septal T1 (ms)	998 ± 112	1012 ± 136	986 ± 85	0.32
Septal T2 (ms)	57 ± 5	57 ± 4	57 ± 5	0.64
Left atrial volume (mL/m^2^)	33.6 ± 10.4	31.9 ± 11.2	35.0 ± 9.6	0.17
LV E/A ratio	1.1 ± 0.4	1.1 ± 0.5	1.1 ± 0.3	0.70
LV E/é ratio	11.4 ± 3.9	13.0 ± 4.6	9.9 ± 2.3	< 0.001

Group comparison of magnetic resonance and echocardiography data. Continuous data are shown as mean ± SD and categorical data as n (%). *E* early diastolic velocity of mitral inflow, *A* late diastolic velocity of mitral inflow, *é* early diastolic mitral annular velocity

In 15 cases, septal T1 and T2 could not be adequately quantified due to image quality issues and were therefore excluded in the statistical analyses. For native T1, ICC for intra- and interreader agreement was 0.998 and 0.996, respectively.

### Circulating cardiovascular biomarkers

For all four Bayesian models, predictors that were at least *substantial* (posterior inclusion probability > 0.76) were as follows:

- IL-1RT2 (interleukin-1 receptor type 2) was a *strong* positive predictor for LV concentricity.
- Leptin was a *strong* negative predictor for both LV mass and LV end-diastolic volume.
- BNP (brain natriuretic peptide) was a *strong* positive predictor for LV mass, and a *substantial* positive predictor for LV end-diastolic volume.

For LV E/é, no predictors passed the *substantial* cut-off in posterior inclusion probability.

### Relationship between liver characteristics and cardiac remodeling

ALT was significantly correlated to LV concentricity (*R* = 0.23, *p* = 0.029). GGT was significantly correlated to LV end-diastolic volume (R = −0.25, *p* = 0.017), LV concentricity (R = 0.31, *p* = 0.0030), and LV E/é ratio (R = 0.435, *p* < 0.001).

**Fig. 4:**
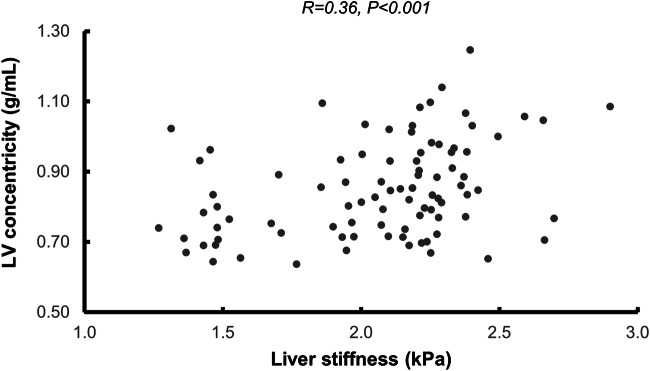
Scatterplot of LV concentricity (Y-axis) and liver stiffness (X-axis), with corresponding Pearson correlation coefficient and *p* value

Over quartiles of liver stiffness, there was an increase in LV concentricity (*p* = 0.0032) (Table 3, Fig. 4). Over quartiles of liver PDFF, there was a decrease in LV end-diastolic volume (*p* = 0.0025), an increase in LV concentricity (*p* = 0.0017), and an increase in LV E/é ratio (*p* = 0.015) (Table 4).

**Table 3:** Quartiles of liver stiffness

	Liver stiffness				
quartile	1	2	3	4	*p* value
mean kPa (min–max)	1.5 (1.3–1.9)	2.0 (1.9–2.2)	2.2 (2.2–2.3)	2.4 (2.3–2.9)	
LV ejection fraction (%)	58 ± 9	60 ± 6	58 ± 11	61 ± 7	0.40
LV mass (g/m^2^)	58 ± 11	60 ± 10	60 ± 11	64 ± 12	0.26
LV end-diastolic volume (mL/m^2^)	75 ± 19	74 ± 14	70 ± 11	70 ± 17	0.59
LV concentricity (g/mL)	0.79 ± 0.13	0.82 ± 0.10	0.86 ± 0.13	0.94 ± 0.15	0.0032
LV stroke volume (mL/m^2^)	43 ± 8	44 ± 8	40 ± 9	42 ± 10	0.34
LV E/é ratio	10.6 ± 3.2	11.3 ± 4.1	12.2 ± 4.3	11.8 ± 4.2	0.64
Septal T1 (ms)	1010 ± 121	989 ± 113	999 ± 119	1003 ± 102	0.79
Septal T2 (ms)	57 ± 5	56 ± 4	57 ± 4	59 ± 5	0.30

Comparison of cardiac imaging parameters between quartiles of liver stiffness. Unless otherwise specified, data is shown as mean ± SD. *p* values were derived from the Kruskal–Wallis test

**Table 4:** Quartiles of liver PDFF

	Liver PDFF				
quartile	1	2	3	4	*p* value
mean % (min-max)	1% (1–2%)	4% (2–6%)	9% (6–12%)	21% (12–35%)	
LV ejection fraction (%)	58 ± 6	58 ± 8	59 ± 8	62 ± 9	0.16
LV mass (g/m^2^)	61 ± 10	62 ± 14	59 ± 9	61 ± 12	0.93
LV end-diastolic volume (mL/m^2^)	78 ± 15	77 ± 14	69 ± 15	68 ± 17	0.025
LV concentricity (g/mL)	0.79 ± 0.12	0.80 ± 0.13	0.87 ± 0.11	0.92 ± 0.14	0.0017
LV stroke volume (mL/m^2^)	45 ± 9	44 ± 8	40 ± 7	41 ± 10	0.11
LV E/é ratio	9.8 ± 2.6	10.7 ± 3.7	12.2 ± 4.8	12.9 ± 3.8	0.015
Septal T1 (ms)	998 ± 56	973 ± 112	1021 ± 135	1002 ± 139	0.84
Septal T2 (ms)	56 ± 4	59 ± 6	56 ± 4	57 ± 4	0.28

Comparison of cardiac imaging parameters between quartiles of liver PDFF. Unless otherwise specified, data is shown as mean ± SD. *p* values were derived from Kruskal-Wallis test

In a multiple linear regression analysis, liver stiffness was positively associated with LV concentricity (Beta = 0.26, *p* = 0.0053), independently of liver PDFF and diabetes. This association was attenuated (Beta = 0.17, *p* = 0.077), when also adjusting the model for levels of IL-1 RT2 (Table 5).

**Table 5:** Multiple linear regression

Model A	Left ventricular concentricity (g/mL)		
	Standardized Beta	*p* value	model *R*^2^
Liver stiffness (kPa)	0.26	0.0053	**0.319**
Liver fat (log%)	0.21	0.037	
Group (diabetes)	0.31	0.0028	

Model B	Left ventricular concentricity (g/mL)		
	Standardized Beta	*p* value	model *R*^2^
Liver stiffness (kPa)	0.17	0.077	**0.394**
Liver fat (log%)	0.13	0.19	
Group (diabetes)	0.27	0.0075	
IL-1RT2 (2^NPX^)	0.30	0.0034	

Two multiple linear regression models with left ventricular concentricity as the dependent variable. Liver fat percentage was logarithmically transformed. For the group variable, diabetes participants were coded as 1 and controls as 0. Beta coefficients were standardized

## Discussion

In this study, using advanced magnetic resonance techniques, we found that a majority of the participants displayed hepatic steatosis. NAFLD is likely the predominating etiology for these cases, as only one participant reported excessive alcohol consumption, and due the fact that NAFLD is the dominant cause of chronic liver disease in the Swedish population.

In spite of the high proportion of subjects with hepatic steatosis, liver stiffness values were not suggestive of advanced liver fibrosis in any participant. To the best of our knowledge, there are no published reference values on liver fibrosis stages, for the specific 3D MRE method with a transducer frequency of 56 Hz used in the current study. However, previous studies using 60–65 Hz find cut-off values for cirrhosis ranging from 4.1 to 4.4 kPa [32, 33], which is more than 1 kPa *above* the highest value of 2.9 kPa observed in our cohort. We believe this gap is too large to be explained by different MRE settings, thus still suggesting the absence of advanced liver fibrosis among our subjects. Nonetheless, stiffness in the liver was related to increased LV concentricity, independently of liver fat and diabetes. This association was attenuated when also adjusting for circulating levels of IL-1 receptor type 2, the biomarker with the strongest association to LV concentricity in the Bayesian statistical analysis.

### Pre-cirrhotic liver fibrosis and cardiac remodeling

The term ‘liver-heart axis’ [8], has been used to describe the phenomenon where the diseased liver adversely affects the heart. In liver cirrhosis, patients are prone to developing ‘cirrhotic cardiomyopathy’, a distinct cardiac phenotype characterized by impaired systolic response to stress, resting diastolic impairment and QT interval prolongation [34]. With the emerging ability to study cardiac and hepatic tissues through magnetic resonance techniques, several recent MRI studies have linked cirrhotic cardiomyopathy to a concurrent increase in diffuse myocardial fibrosis [8, 9], a process that appears to reverse when cirrhosis is treated with liver transplantation [10].

However, only two previous studies have specifically investigated the relationship between LV remodeling and liver stiffness in non-cirrhotic individuals. Using ultrasound-based liver elastography, Lee et al [6] studied patients with and without NAFLD and showed that both liver fat as well as liver stiffness was associated with higher resting LV E/é. Canada et al [7] found in 36 subjects with NAFLD that increasing histological severity of liver fibrosis was associated with lower peak oxygen uptake during exercise, higher LV E/é during exercise, and slower heart rate recovery after exercise, but no relationship was found to resting LV E/é. Notably, both of these studies used echocardiography to determine cardiac structure and function, and neither study reported any clear relationship between cardiac structure and liver fibrosis.

Compared to earlier works, our current study provides several significant contributions to the so far limited literature on early liver fibrosis and LV remodeling, as MRI studies on participants from the general population are lacking [35]. As Canada et al and Lee et al could only demonstrate relationships to diastolic function, our more accurate assessment of cardiac morphology from cardiac MRI, add new insights into the specific structural aspects of LV remodeling in relation to liver fibrosis. Moreover, we used magnetic resonance techniques also for the assessment of liver characteristics. This method enables high-precision and noninvasive measurement of both liver fat and liver stiffness and outperforms ultrasound techniques, at least in terms of liver fat quantification [12]. Although liver biopsy is regarded as the best standard method to assess liver fibrosis [36], the limited number of subjects in the study by Canada et al may reflect the high cost and invasiveness associated with this method. Last, our extensive analysis of circulating cardiovascular biomarkers, as discussed below, also has the potential to provide new mechanistic understandings of the relationship between liver fibrosis and LV remodeling.

Although concentric LV remodeling was independently related to both liver fat and fibrosis, we did not find any evidence of concurrent alterations in native septal T1 or T2. One possible explanation is that LV remodeling in this setting is not followed by any simultaneous change in myocardial tissue characteristics, such as diffuse myocardial fibrosis. Another perhaps more likely explanation is that potential myocardial tissue alterations are too subtle to be detected by native T1 mapping. When Kim et al found that a significant decrease in myocardial extracellular volume (ECV) fraction occurred after liver transplantation, they observed no changes in native T1 [10]. This supports the fact that alterations in myocardial tissue characteristics could be detected through, for example, ECV quantification but not by native T1 mapping.

### Mechanistic interlinks and circulating cardiovascular biomarkers

To explore possible mechanistic interlinks between cardiac remodeling and the liver, we analyzed cardiovascular biomarkers of inflammation and fibrosis and found that the association between liver stiffness and LV concentricity was attenuated when adjusting for circulating levels of interleukin-1 receptor type 2 (IL-1RT2). This could indicate a mechanistic role of IL-1RT2 in concentric LV remodeling. IL-1RT2 is a soluble decoy receptor that negatively regulates the inflammatory activity of IL-1β [37]. Circulating levels of IL-1RT2 have previously been associated with long-term adverse cardiac remodeling. Orrem et al [38] found that levels of IL-1RT2 were elevated in ST-elevation myocardial infarction and correlated with higher myocardial infarct size and long-term adverse LV remodeling, including lower LV ejection fraction four months after infarction. The authors proposed that, as IL-1RT2 negatively regulates IL-1β stimuli, it might counteract the beneficial inflammatory response necessary for infarct healing and adaptive LV remodeling. Similar to Orrem et al, we did not find any associations to LV remodeling for IL-1 receptor antagonist protein or IL-1 receptor type 1.

Though the structural and functional cardiac alterations are likely to have a different underlying nature in liver disease compared to in myocardial infarction, it appears at least plausible that IL-1RT2 is related to cardiac remodeling. Moreover, as IL-1 family cytokines have been shown to play an important role for hepatic inflammation and fibrosis progression in NAFLD [39, 40], the role of IL-1 cytokines as a mechanistic interlink between liver fibrosis and LV remodeling is suggestive and may warrant further research.

### Limitations

The current study poses considerable strengths in the study of LV remodeling and liver disease, such as the use of magnetic resonance techniques for the assessment of both liver and heart, as well as the extensive number of analyzed cardiovascular biomarkers. However, some limitations need to be mentioned. First, as no liver biopsies were performed on the participants in this study, we are unable to determine histopathological inflammation in the liver. Second, no external contrast agent was administered during the magnetic resonance examinations, and we are therefore unable to assess late gadolinium enhancement and ECV in the myocardium. Last, further research on larger study populations could improve the generalizability of the findings.

## Conclusions

In the current study, advanced magnetic resonance imaging was used to bring novel insights into the liver-heart axis in participants from the general population. The results show that liver fibrosis is independently associated with concentric LV remodeling, a relationship that may involve IL-1 signaling. Future studies could establish causal relationships between liver fibrosis and LV remodeling, as well as provide further understanding of the role of IL-1 signaling in this clinical context.

## Supplementary information

Electronic Supplementary Material

## Abbreviations

A: Late diastolic velocity of mitral inflow
ALT: Alanine aminotransferase
BMI: Body mass index
BSA: Body surface area
E: Early diastolic velocity of mitral inflow
é: Early diastolic mitral annular velocity
GGT: Gamma glutamyl transferase
HDL: High density lipoprotein
IL-1RT2: Interleukin 1 receptor type 2
LV: Left ventricular
MRE: Magnetic resonance elastography
MRI: Magnetic resonance imaging
MRS: Magnetic resonance spectroscopy
NAFLD: Non-alcoholic fatty liver disease
PDFF: Proton density fat fraction
SCAPIS: Swedish CArdioPulmonary bioImage Study
SSVS: Stochastic search variable selection
T2D: Type 2 diabetes
