# Supplementary material for: Liver fibrosis is associated with left ventricular remodeling: insight into the liver-heart axis
Source: Eur Radiol. 2024 May 25;34(11):7492–502. doi: 10.1007/s00330-024-10798-1 (PMC11519090; doi:10.1007/s00330-024-10798-1)
Supplement: Supplementary file 1 — Electronic Supplementary Material [file 330_2024_10798_MOESM1_ESM.pdf]

Liver fibrosis is associated with left ventricular remodeling: Insight into the liver-heart axis

Electronic Supplementary Material (ESM)

Eur Radiol (2024) Edin C, Ekstedt M, Karlsson M et al.

Supplementary material

1 Magnetic resonance sequences

1.1 Magnetic resonance elastography (MRE) eXpresso sequence:
The 3D-MRE method has been described previously in [*Forsgren, Mikael F et al. "Comparing hepatic 2D and 3D magnetic resonance elastography methods in a clinical setting - Initial experiences." European journal of radiology open vol. 2 66-70. 28 Apr. 2015, doi:10.1016/j.ejro.2015.04.001*]. Briefly, an active electrodynamic transducer (Philips Medical) was used to transmit mechanical waves at 56 Hz, on the right side of the patient at the level of the liver, with the patient in the supine position. Images were then acquired in four separate breath-holds, and nine slices were acquired with a slice thickness of 4 mm each. The mean shear elasticity was reported in units of kPa (at the mechanical vibration frequency) for the right liver lobe.

1.2 Liver proton magnetic resonance spectroscopy (^{1}H-MRS) PRESS sequence:
Sequence settings: TR=1500 ms, TE=35 ms, volume of interest [VOI]= c. 30x30x30 mm3, NSA=8.

1.3 Balanced steady state-free precession
Sequence settings: TE=1.4 ms, TR=2.7-2.8 ms, flip angle=50°, SENSE factor 2 x 2, spatial resolution 2.5x2.5x8.0 mm^3, acquired temporal resolution 49-69 ms, breath hold duration 16 sec, reconstructed spatial resolution 1.0x1.0x8.0 mm^3, reconstructed heart phases 30.

1.4 3D-QALAS
The 3D-QALAS (3D-quantification using an interleaved Look-Locker acquisition sequence with T2 preparation pulse) consists of 5 segmented cardiac-triggered 3D gradient echo acquisitions which are acquired in parallel. Sequence settings were TE=1.5 ms, TR=3.0 ms, repeated 72 times, Read-out time 215 ms, set to late diastole. Flip angle=4°. The FOV was 350x350 mm oblique with 13 slices of 6 mm. Prior to the first acquisition an R2-sensitizing phase was applied, consisting of a hard 90 degrees RF pulse, 4 adiabatic 180 degrees refocusing pulses and a hard -90 pulse (TE = 100 ms). Prior to the second acquisition an R1 sensitizing phase was applied, consisting of an adiabatic inversion pulse. The scan time was a single breath-hold of 15 heart beats.

2 Echocardiography
The echocardiographic exams in SCAPIS Linköping were performed using GE Vivid E95 rev 201 or E9 (GE Vingmed Ultrasound). From an apical position, B-mode cine loops were acquired at a framerate > 40 frames per second together with pulsed-wave blood Doppler as well as tissue Doppler of wall velocity. Software GE EPPC SWO v201 (GE healthcare) was used to analyze all data. Diastolic functional parameters included in this study were: ratio of left ventricular early diastolic filling (E) to late diastolic filling (A) and early mitral annular velocity (e´), respectively, as well as left atrial volume.
